# On the Origin of the 1,000 Hz Peak in the Spectrum of the Human Tympanic Electrical Noise

**DOI:** 10.3389/fnins.2017.00395

**Published:** 2017-07-11

**Authors:** Javiera Pardo-Jadue, Constantino D. Dragicevic, Macarena Bowen, Paul H. Delano

**Affiliations:** ^1^Departamento de Neurociencia, Facultad de Medicina, Universidad de Chile Santiago, Chile; ^2^Departamento de Fonoaudiología, Facultad de Medicina, Universidad de Chile Santiago, Chile; ^3^Departament of Linguistics, Australian Hearing Hub, Macquarie University Sydney, NSW, Australia; ^4^Departamento de Otorrinolaringología, Hospital Clínico de la Universidad de Chile Santiago, Chile

**Keywords:** electrocochleography, round window noise, tympanic membrane, spontaneous activity, auditory nerve, vestibular nerve

## Abstract

The spectral analysis of the spontaneous activity recorded with an electrode positioned near the round window of the guinea pig cochlea shows a broad energy peak between 800 and 1,000 Hz. This spontaneous electric activity is called round window noise or ensemble background activity. In guinea pigs, the proposed origin of this peak is the random sum of the extracellular field potentials generated by action potentials of auditory nerve neurons. In this study, we used a non-invasive method to record the tympanic electric noise (TEN) in humans by means of a tympanic wick electrode. We recorded a total of 24 volunteers, under silent conditions or in response to stimuli of different modalities, including auditory, vestibular, and motor activity. Our results show a reliable peak of spontaneous activity at ~1,000 Hz in all studied subjects. In addition, we found stimulus-driven responses with broad-band noise that in most subjects produced an increase in the magnitude of the energy band around 1,000 Hz (between 650 and 1,200 Hz). Our results with the vestibular stimulation were not conclusive, as we found responses with all caloric stimuli, including 37°C. No responses were observed with motor tasks, like eye movements or blinking. We demonstrate the feasibility of recording neural activity from the electric noise of the tympanic membrane with a non-invasive method. From our results, we suggest that the 1,000 Hz component of the TEN has a mixed origin including peripheral and central auditory pathways. This research opens up the possibility of future clinical non-invasive techniques for the functional study of auditory and vestibular nerves in humans.

## Introduction

Auditory nerve fibers (ANF) transmit action potentials from the cochlea to the brain. This neural activity can be recorded spontaneously, -in the absence of acoustic stimulation-, or in response to auditory stimuli (Walsh et al., 1972; Kiang et al., 1976; Manley and Robertson, 1976; Liberman and Kiang, 1978). Dolan et al. (1990) placed an electrode near the round window (RW) of guinea pigs and recorded spontaneous electric activity. The spectral analysis of this signal showed a broad peak centered between 800 and 1,000 Hz. As the extracellular field potentials generated by action potentials of the auditory nerve last 1–2 ms (Kiang et al., 1976), their spectral analysis contributes to the frequency band of this peak. Therefore, these authors suggested that this peak at ~900 Hz reflects the sum of the spontaneous discharge of auditory nerve neurons (Dolan et al., 1990). Since the first recordings of round window noise (RWN) made by Dolan et al. (1990), several authors have studied its properties, including its possible origin (McMahon and Patuzzi, 2002; Searchfield et al., 2004), olivocochlear influence (Popelar et al., 1996; Lima da Costa et al., 1997), and its relationship with tinnitus (Cazals et al., 1998). In a clinical setting, the functional evaluation of the auditory nerve is essential for the perceptual outcome of cochlear implant patients (Abbas et al., 2017). In humans, while stimulus driven auditory-nerve activity can be measured through compound action potentials of the auditory nerve (CAP) or by means of wave I from auditory brainstem responses, the spontaneous activity of ANF can only be recorded during neurosurgical procedures, like cerebellopontine angle surgery (Martin, 1995). However, to date there are no good non-invasive electrophysiological measures of auditory nerve status in profound deaf patients that are candidates for cochlear implantation.

We propose that it is possible to record the tympanic electric noise (TEN) using a non-invasive method, similar to that used for tympanic electrocochleography (ECochG), which could be indicative of auditory-nerve spontaneous activity. The aim of the present work is to analyze the frequency components of the electric noise recorded from the tympanic membrane in humans, and to study whether the amplitude of these frequency components depends on acoustic and vestibular caloric stimulation. We found a reliable frequency peak at ~1,000 Hz in the TEN signal of all subjects recorded in the absence of acoustic stimulation. In addition, we found that in most subjects, the amplitude of the TEN increased with acoustic and caloric vestibular stimulation. The current study demonstrates the possibility to further contributions of the TEN as a potential clinical technique for the functional study of auditory nerve in humans.

## Materials and methods

### Subjects

Twenty-four adults of both sexes (12 women) were included in this study. The mean age was 25.4 ± 4.93 years, ranging between 20 and 45 years old. All subjects had normal hearing thresholds (audiometric thresholds ≤20 dB HL from 250 Hz to 4,000 Hz). This study was carried out in accordance with the recommendations for clinical research of the University of Chile, and was approved by the Institutional committee of Ethics (Hospital Clínico de la Universidad de Chile). All subjects gave written informed consent in accordance with the Declaration of Helsinki.

### TEN recordings

Tympanic ECochG recordings were obtained in awake subjects under silent condition or in response to stimuli of different sensory modalities in a sound-attenuating room. The external ear canal was cleaned with saline solution (0.9% sodium chloride solution) and ear wax was removed by aspiration. Then, a wick electrode (Intelligent Hearing Systems®) was carefully placed on the tympanic membrane. Both procedures were performed by an otolaryngologist under microscopic view. Surface electrodes were placed on the forehead (ground) and on the contralateral ear lobe (reference). The electrodes were secured with tape to the skin. Impedance of reference and ground electrodes were maintained below 5 kΩ, while we tried to keep the impedance of the tympanic electrode below 25 kΩ by means of a conductive gel applied to the tympanic membrane. This conductive gel was used in addition to the recommendation given by the manufacturer of hydrating the wick electrode with saline solution. At the end of the experiments, the conductive gel applied to the tympanic membrane was aspirated by an otolaryngologist under microscopic view. We used a PZ3 preamplifier on the ECochG channel coming from the tympanic electrode (low pass filtered at 10 kHz), and a multiprocessor (RZ6) connected to a computer (Tucker-Davis Technologies®). Both equipments were controlled with a custom software (System 3, Tucker-Davis Technologies®) to record data and generate sounds with sampling rate of 50 kHz.

### Stimulation protocols

Electrophysiological recordings were conducted with the subject lying down on a clinical bed. The TEN signal was recorded during six minutes without any external stimuli. Subjects were asked to remain still and quiet during this time. This protocol was repeated twice to test reliability of recordings. Furthermore, electromyographic and neural activity of trigeminal, facial and oculomotor nerves were explored by means of isometric muscle contractions of masseter, blinking, and ocular movements during TEN recordings. In addition, to investigate the origin of the TEN 1,000 Hz peak, we performed electrocardiographic (ECG) like recordings in the absence of external stimuli, placing an additional surface electrode on the ipsilateral wrist while maintaining ground (forehead) and reference (contralateral ear lobe) electrodes.

### Acoustic stimulation (*n* = 11)

In a subset of the volunteers (*n* = 11) we performed acoustic stimulation presenting an ipsilateral, filtered, and continuous broad-band noise (4–20 kHz). The broad-band noise was digitally generated at 50 kHz sampling rate, and high pass filtered at 4 kHz to avoid the acoustic energy overlapping with the spectrum of the TEN peak at 1 kHz. The noise was delivered by insert phones (ER-10C, Etymotic Research®) at 72 and 82 dB SPL. Phones were previously calibrated by means of 2-ml artificial cavity (up to 10 kHz). The tympanic electrode was placed on the ear drum and fixed to the ear lobe. After that we used a large foam tip to seal the ear canal, which might yield to slightly different sound pressure levels to those measured in the two-ml cavity. The experimental protocol consisted of 40–60 s of spontaneous recording (silent period without stimulation) followed by acoustic stimulation with each intensity sequentially presented for 60–80 s (72 and 82 dB SPL). The TEN signal was recorded continuously during the full protocol. In addition, to confirm adequate impedance of the tympanic electrodes, acoustically evoked CAPs at different sound pressure levels were obtained (100 μs clicks, presented at 21 Hz rate, repetitions = 1,000).

### Vestibular stimulation (*n* = 8)

To stimulate the vestibular nerve fibers, we decided to perform vestibular stimulation with caloric stimuli in eight volunteers (*n* = 8). We used bithermal caloric stimulation during TEN recordings (ATMOS Varioair®) at 26°C (cold) and 49°C (warm) delivered during 120 s through the external ear canal. In five subjects, we also tested the effects of 37°C airflow stimulation, as control experiments with body temperature. The auditory and vestibular experiments were performed in different days. The flow of air was delivered to the tympanic membrane by a plastic tube tip connected to the irrigation handle and inserted into the ear canal. In order to confirm vestibular stimulation, the presence of nystagmus was explored using Frenzel goggles (ICS FL-15, Otometrics®). The experimental vestibular protocol consisted in 60 s of spontaneous baseline recording followed by 120 s of caloric stimulation and a final recovery period of 60 s without stimuli. The TEN signal was recorded continuously during the full 240 s protocol.

### Data analysis

Fast Fourier transforms (FFT) were applied to data using time windows of 1,000 ms (1 Hz resolution), which were moved in steps of 800 ms (100 ms overlap each side). This procedure yields a matrix of data corresponding to the time spectrogram of the TEN signal. Given the small amplitudes of the frequency components of the TEN signal (tens of nanoVolts), an iterative smoothing algorithm for removing the noise peaks at 50 Hz and its harmonics was developed. First, a smoothing boxcar function using 35 Hz width (21 points) was applied to the original average spectrum of the TEN signal. Then, the original average spectrum was compared to its smoothed version by subtraction. All differences (point by point) yielded statistical values (mean ± standard deviation) which were used to detect outlier points. Every point (and both immediate neighboring points) with a difference exceeding mean ± three standard deviations were eliminated from the original average spectrum. This procedure was iterated three times to provide a satisfactory denoised spectrum, as judged by visual inspection. Finally, the missing points of the denoised spectrum were filled by linear interpolation. With the denoised version of each TEN average spectrum (taken from the 7 consecutive spectra inside 6 s), we could automatically calculate integral values between 650 and 1,200 Hz from the amplitude spectrum. The magnitude of the peak between 650 and 1,200 Hz of the TEN, as measured by this integral in 6 s steps, was the *sample* in statistical analyses during auditory and vestibular stimulation (SigmaPlot 12.5, Systat Software®, Inc., USA). In the acoustic and vestibular stimulation experiments, data were expressed as dB of change from baseline levels (dB = 20^*^LOG x/baseline amplitude). The normal distribution of these samples were evaluated using Shapiro-Wilk tests. If the distribution was normal, possible differences between conditions were evaluated with one-way ANOVA, if not, the Mann-Whitney or Kruskal-Wallis tests were applied, depending of the number of conditions analyzed. In every case, a *p* < 0.05 was considered as a significant difference.

## Results

**Figure 1:**
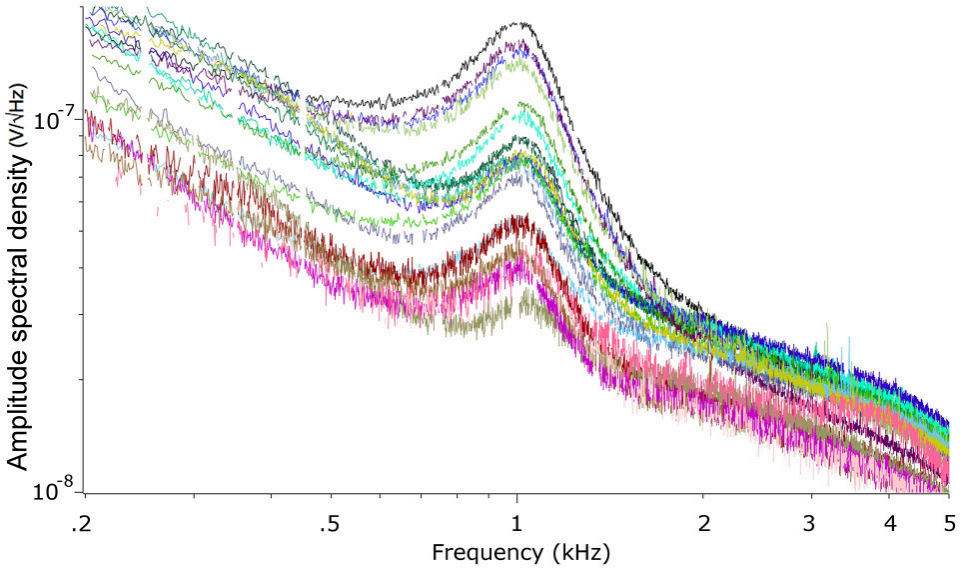
Power spectrum of the tympanic electric noise. Each colored curve represents one different subject. Note the presence of a broad peak in all subjects around 1,000 Hz. The missing points in the curves were eliminated by the denoise procedure (described in the Methods section).

**Figure 2:**
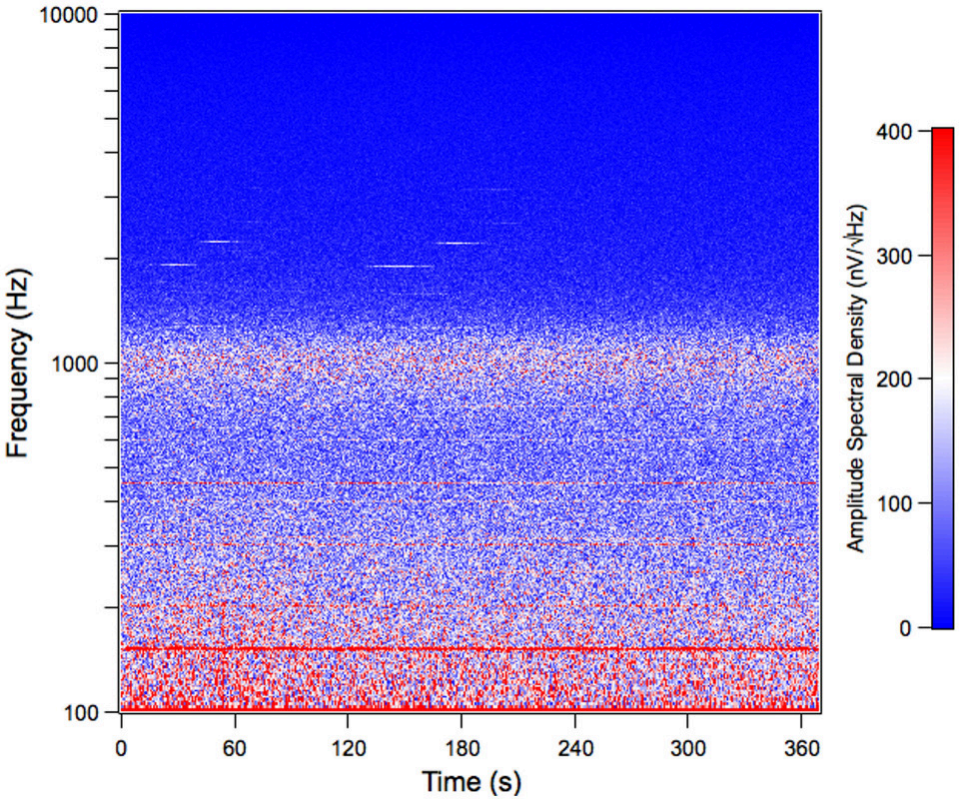
Time spectrum of the tympanic electric noise in humans. This spectrogram shows the stability of the 1,000 Hz peak throughout a complete session of tympanic electric noise recording (360 s) in one subject. Notice that, although the spectral peak is centered around 1,000 Hz, at the single epoch level (each dot in the time spectrum), varies between 800 Hz and 1,200 Hz. This figure shows data before the denoise procedure. The subject corresponds to the volunteer with the largest peak at 1,000 Hz in Figure 1.

**Figure 3:**
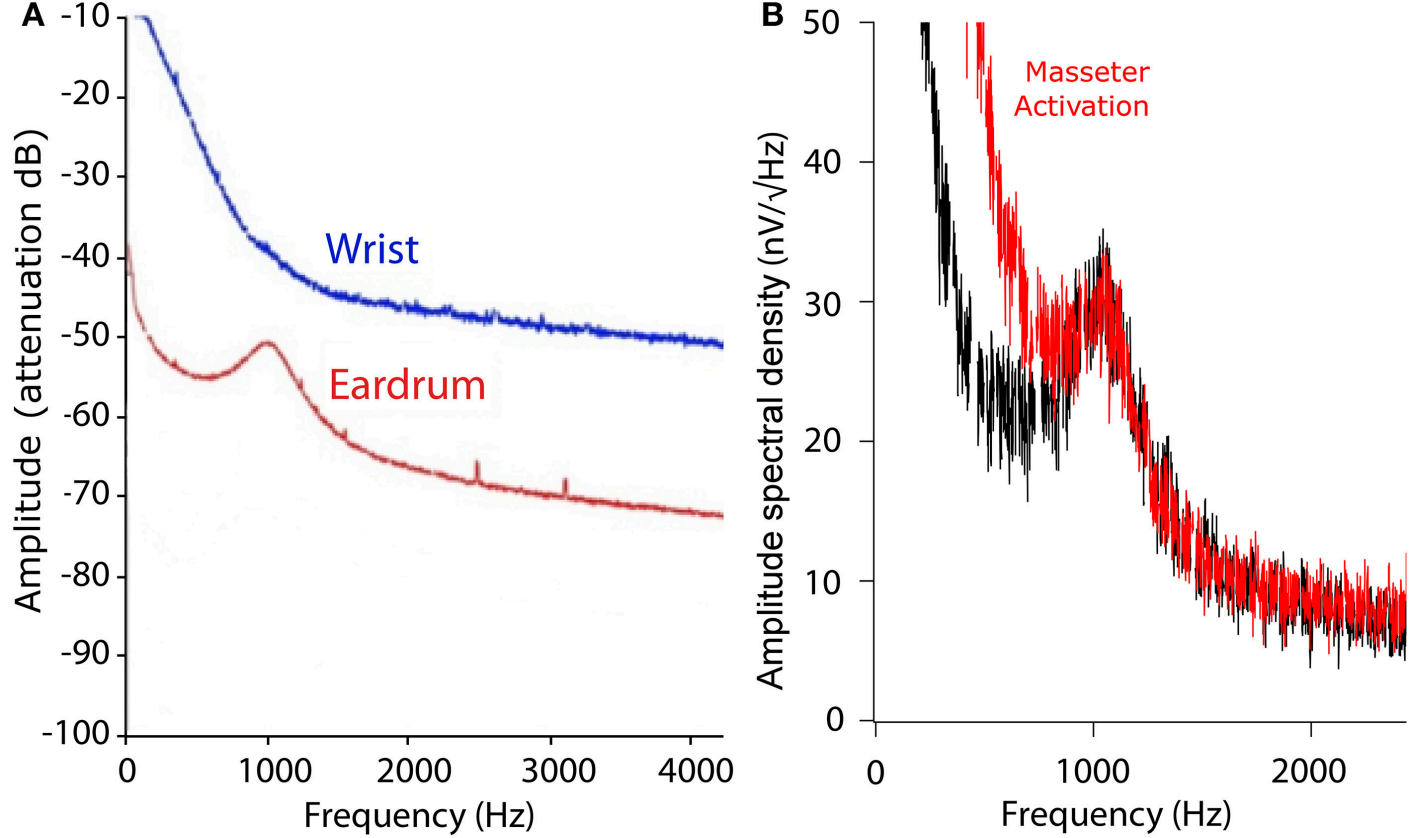
Tympanic electric noise ECG-like and EMG controls. **(A)** Comparison between TEN and ECG-like spectrums. The blue line shows the averaged spectrogram of a 360 s recording from an ECG-like signal with wrist electrodes, while the red line shows the spectrum of the TEN signal in the same volunteer. To compare the wrist and eardrum noise signal, the y-axis is shown in arbitrary units measured in dB of attenuation. The frequency components of the ECG-like signal are probably observed in the low frequency band (<100 Hz) of the corresponding power spectrum. **(B)** TEN spectrum with (red) and without (black) masseter muscle activation. Volunteers activated their masseter muscles through isometric contraction, with mouth closed during 1 min. Note that the muscle activation produces a power increase in the frequency band <800 Hz, but not in the 1,000 Hz peak, probably related to EMG activity.

We recorded the TEN under silent conditions in 24 normal-hearing subjects. All volunteers showed spontaneous activity with a broad spectral peak around 1,000 Hz (Figure 1). The amplitude values of the 1,000 Hz peak of the TEN varied from 5 to 80 nV across subjects. Figure 2 displays an example of time-frequency analysis of the peak at 1,000 Hz in one volunteer in the absence of acoustic stimulation, showing that the 1,000 Hz peak of the TEN remained stable during the 360 s session. Next, to test possible biological artifacts affecting the 1,000 Hz peak from TEN, different control experiments were performed: (1) electrocardiogram like recording, (2) masticatory isometric muscle contractions, (3) blinking, and (4) ocular movements. The 1,000 Hz peak was absent when using a wrist ECG-like electrode configuration, while masseter muscle activation produced an increase in the power of frequencies below 800 Hz, but not in the 1,000 Hz component (Figure 3). Non-significant differences were found during blinking or ocular movements (data not shown).

**Figure 4:**
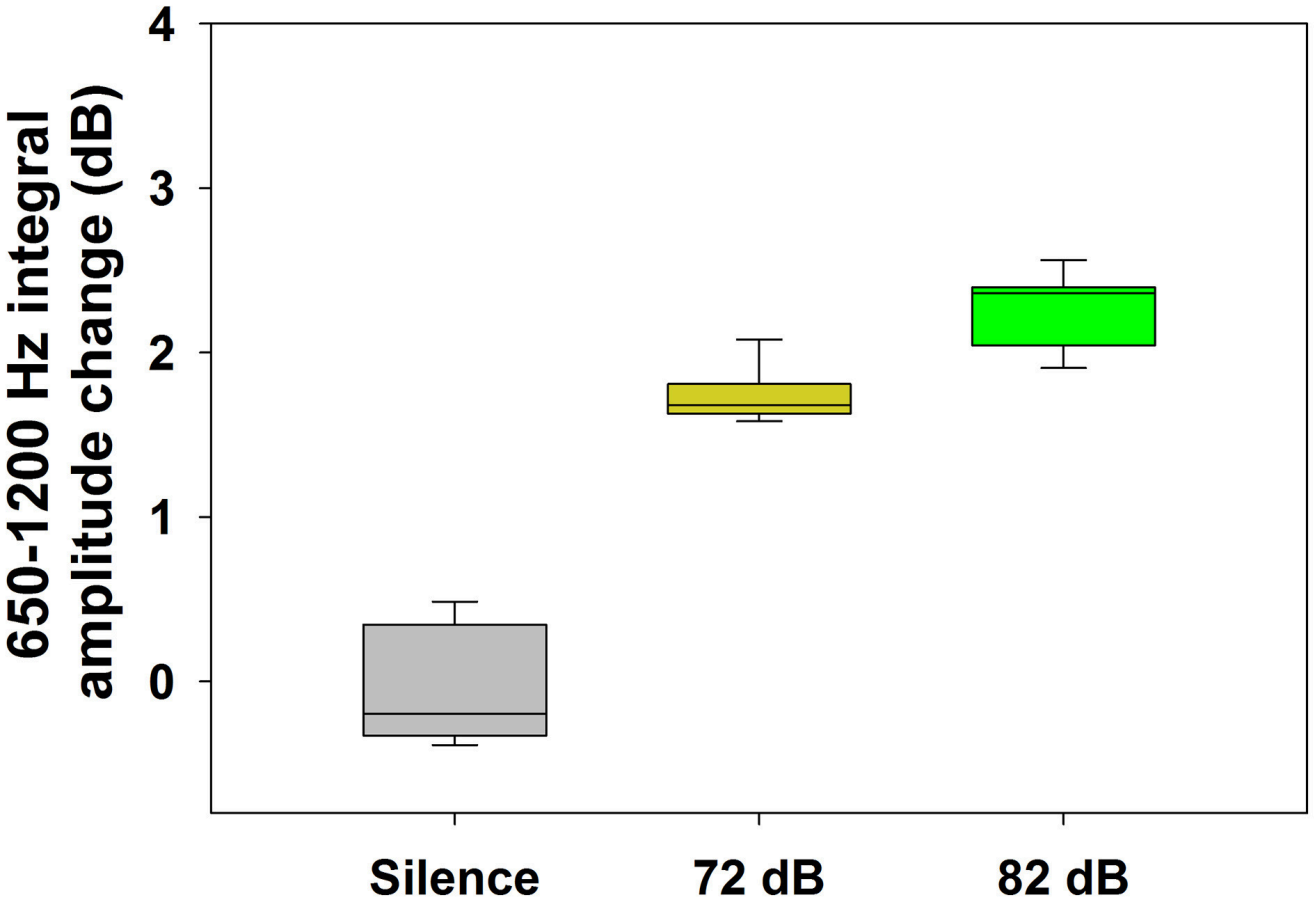
Increase of the TEN 1,000 Hz peak amplitude during auditory stimulation with broad-band noise in the majority of the subjects (*n* = 9). This graph shows the effect (in dB) of broad-band noise stimulation on the nine subjects with an increase in the amplitude of the TEN 1,000 Hz peak (measured as the integral value between 650 and 1,200 Hz) [One way ANOVA, *F*_(2)_ = 241.420, *p* < 0.001; Tukey *post-hoc, p* < 0.05 in the three pairwise comparisons]. In addition to the amplitude increase of the TEN 1,000 Hz peak observed in these nine subjects, in two cases we found an amplitude decrease with broad-band noise stimulation.

**Figure 5:**
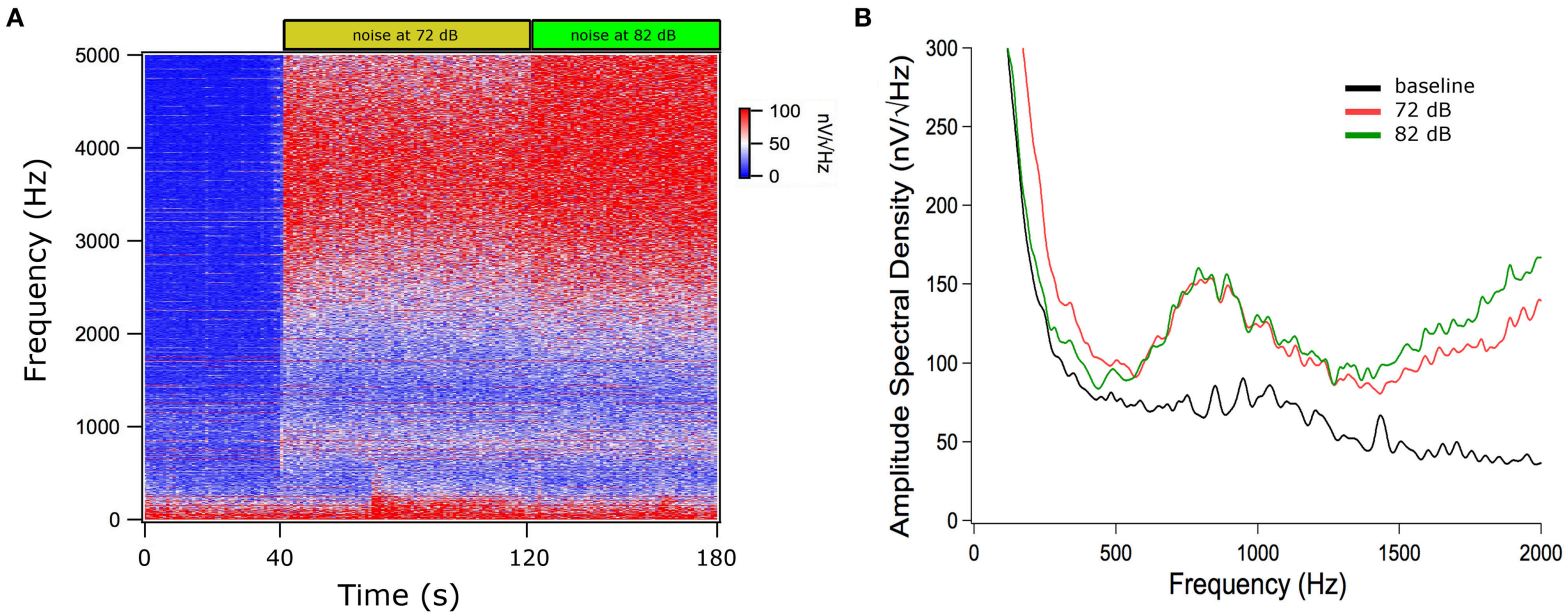
Time and power spectrums of the tympanic electric noise with broad-band noise stimulation. **(A)** Time spectrum, the filtered broad-band noise (>4 kHz) increases the TEN 1,000 Hz peak at 72 and 82 dB. Notice the presence of a cochlear microphonic component above 2 kHz with broad-band noise stimulation. **(B)** Spectrum of the averaged signals in silence (baseline) and with 72 and 82 dB SPL. This figure shows data before the denoise procedure.

Regarding auditory stimulation, we found a significant increase of the TEN 1,000 Hz peak amplitude using ipsilateral broad-band noise in 9 out of 11 subjects at 72 and 82 dB SPL [one way ANOVA, *F*_(2):_ 241.420; *p* < 0.001, Tukey *post-hoc p* < 0.05 compared to silent conditions] (Figure 4). The amplitude increase of the 1,000 Hz peak with 72 dB SPL was 1.74 ± 0.16 dB (mean ± standard deviation); while for 82 dB SPL was 2.27 ± 0.22 dB. In the other two subjects, we found an amplitude reduction with ipsilateral broad-band noise at 72 dB SPL of −0.92 ± 0.25 dB and at 82 dB of −1.06 ± 0.35 dB. Figure 5 shows an example of the time frequency spectrum measured during acoustic stimulation with broad-band noise at 72 and 82 dB SPL. A cochlear microphonic response above 2 kHz is clearly seen in the time spectrum, while an increase of the spectral components of the TEN around 800 Hz is also observed.

**Figure 6:**
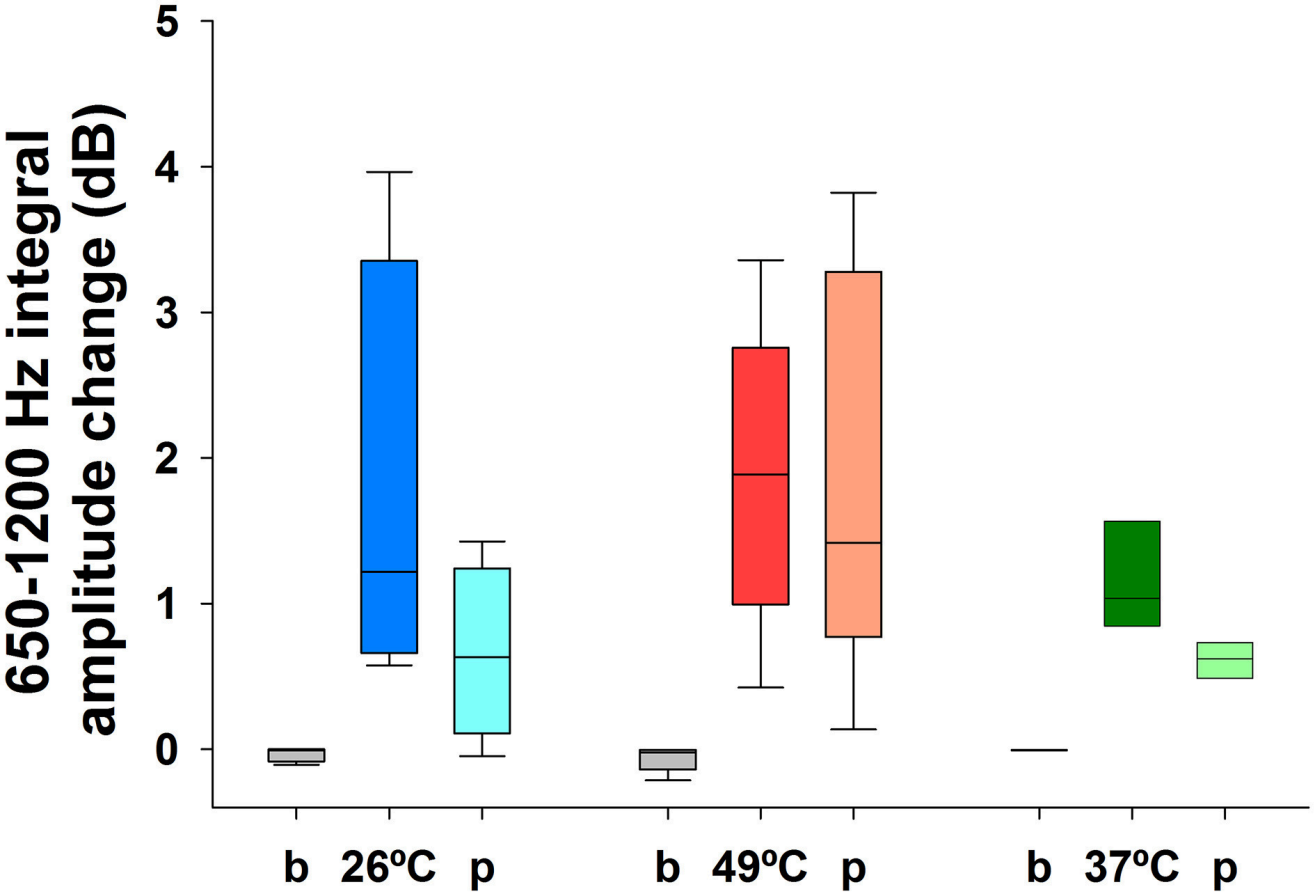
Increase of the TEN 1,000 Hz peak during vestibular caloric stimulation at 26°C (blue), 49°C (red), and 37°C (green). This figure show box-plots of TEN 1,000 Hz peak amplitudes for baseline and caloric stimulation at 26 and 49°C in eight volunteers and at 37°C for three subjects, showing the effect in dB of change (measured as the integral value between 650 and 1,200 Hz). Note that the amplitude of the 1,000 Hz peak of TEN does not return to base levels after warm stimulation at 49°C. [Cold air: Kruskal–Wallis analysis, *H*_(2)_ = 6.038, *p* = 0.037, Dunn *post-hoc* test *p* < 0.05; warm air: Kruskal-Wallis analysis, *H*_(2)_ = 9.420, *p* = 0.009]. Stimulation at 37°C also produced an increase of the 1,000 Hz peak of the TEN (b, baseline and p, recovery period).

**Figure 7:**
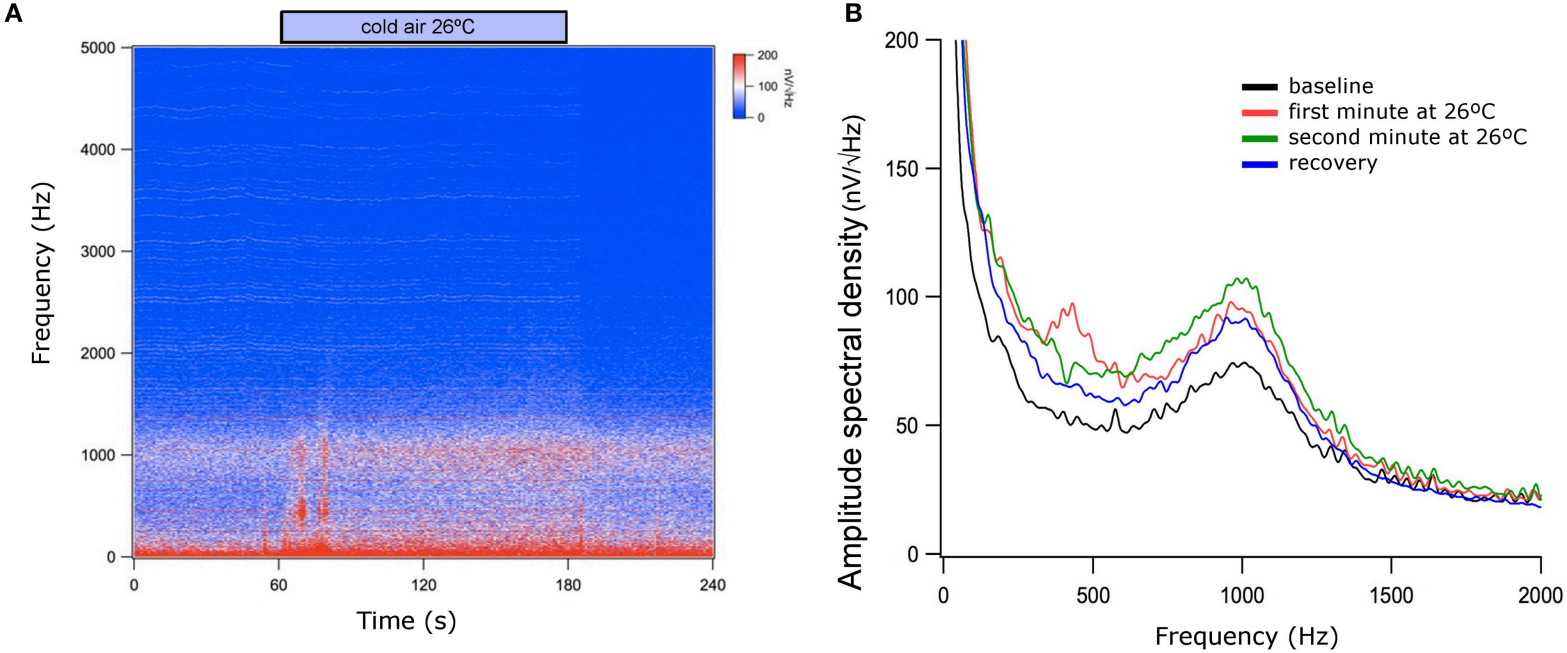
Time and power spectrums of the tympanic electric noise with cold air stimulation at 26°C. **(A)** Notice the presence of low frequency artifacts at the beginning and at the end of the caloric stimulation (around 60 and 180 s), while there is a progressive increase of the 1,000 Hz peak during vestibular stimulation (between 60 and 180 s). **(B)** Spectrum of the averaged signals during baseline period, during the first and second minutes of caloric stimulation and after cold stimulation. Notice that the largest 1,000 Hz peak was obtained in the second minute of cold stimulation. This figure shows data before the denoise procedure.

Vestibular stimulation with cold airflow at 26°C produced an increase in TEN 1,000 Hz peak amplitude (1.74 ± 1.53 dB, mean ± *SD*) which almost returned to base levels (0.66 ± 0.60 dB) during the recovery period [Kruskal–Wallis analysis, *H*_(2)_ = 6.038, *p* = 0.037, Dunn *post-hoc* test *p* < 0.05] (Figure 6). Warm stimulation at 49°C produced a significant increase in the TEN 1,000 Hz peak amplitude (1.89 ± 1.1 dB) compared to base levels. This effect did not return to base levels (1.90 ± 1.4 dB of change) [Kruskal–Wallis analysis, *H*_(2)_ = 9.420, *p* = 0.009]. Figure 7 shows an example of the time frequency spectrum measured during caloric stimulation with air at 26°C. A progressive increase of the TEN 1,000 Hz peak is observed along cold stimulation. Next, we measured the effects of 37°C airflow stimulation in the ear canal in five subjects as a control condition. Since two of the five subjects had nystagmus and vertigo with this temperature, we describe the effects of this stimulation in three volunteers. A similar increase to those observed in cold stimulation was obtained in these subjects (37°C: 1.15 ± 0.37 dB; recovery 0.61 ± 0.12 dB) (Figure 6).

## Discussion

We found a reliable frequency peak at ~1,000 Hz in the spectral analysis of the TEN measured with a non-invasive technique in humans. In the majority of the cases, the amplitude of the TEN 1,000 Hz peak increased with auditory and vestibular stimulation, but not with motor activation.

### Differences between RWN and TEN

The RWN studied in animal models has received a number of different names: ensemble spontaneous activity (Snyder and Schreiner, 1987), ensemble spontaneous neural activity (Martin et al., 1993), ensemble background activity (Popelar et al., 1996), average spectrum of electrophysiological cochlear activity (Cazals and Huang, 1996), and spontaneous neural noise (McMahon and Patuzzi, 2002). These studies were all performed with electrodes located near the round window membrane, they all showed an energy peak between 800 and 1,000 Hz in silent conditions, and probably correspond to the same biological signal. These animal studies have shown that the RWN is a biological signal recorded in silent conditions that disappears in post-mortem status (Dolan et al., 1990), and that the spectral peak found between 800 and 1,000 Hz probably corresponds to the extracellular field potentials generated by action potentials of the ANFs (Kiang et al., 1976; Versnel et al., 1992; Searchfield et al., 2004). In guinea pigs, the contribution of auditory-nerve action potentials to the RWN peak at 800–1,000 Hz was demonstrated by applying to the inner ear pharmacological treatments that block or reduce the neural activity (Searchfield et al., 2004). They showed that the ~900 Hz peak was reduced in amplitude or disappeared like in post-mortem animal recordings. Furthermore, these studies suggested that the generation or principal contribution of the RWN is given by the spontaneous activity of ANFs arising from the basal cochlear region and consequently, the amplitude of the RWN peak correlates with good auditory sensitivity at high frequencies (Dolan et al., 1990; Searchfield et al., 2004). Together, these studies performed in animal models evidence that the spectral peak ~900 Hz of the neural noise recorded near the round window is an indirect measure of the ANF spontaneous activity.

In the present work, we recorded the electrical noise from the tympanic membrane in human subjects, and obtained a similar broad frequency peak (around 1,000 Hz). Although, the tympanic membrane is relatively close to the inner ear, the anatomic location of the positioned electrode is different from that of animal models (round window membrane). The consequence of the difference in the recording position is that neural contributors can be different and therefore the ~900 Hz frequency peak obtained from the RWN in the animal models cannot be directly equated to the TEN 1,000 Hz peak in humans. For this reason, in addition to the auditory and vestibular nerves, we conducted experiments to rule out possible contributions of cranial nerves passing near the tympanic membrane, as facial, and trigeminal nerves. As we found no amplitude changes of the TEN 1,000 Hz peak with ocular movements, or facial and masticatory tasks, we focused on auditory and vestibular stimulations.

### TEN 1,000 Hz peak and auditory stimuli

In the absence of acoustic stimulation, we found a repeatable frequency peak at ~1,000 Hz in the TEN signal of all recorded subjects. One possibility is that the spontaneous 1,000 Hz peak in the TEN is driven by ANF responses to self-generated sounds, but it could also reflect non-stimulus driven spontaneous activity. Independently of its acoustic source, this neural peak might be used as an additional objective measure of cochlear nerve function, with the advantage of providing a measure of non-synchronized activity to auditory stimuli.

In addition, amplitude changes of the TEN 1,000 Hz peak were clearly obtained with acoustic stimulation. We found an amplitude increase of the TEN 1,000 Hz peak with broad-band noise in 9 out of 11 volunteers (Figure 4). As we used a high pass filtered noise (4–20 kHz), we stimulated the base of the cochlea, therefore, this increase probably corresponds to recruitment of ANFs innervating the first cochlear turns and not to cochlear microphonic potentials in response to 1,000 Hz (Heil and Peterson, 2015). On the other hand, in two cases we found amplitude reductions of the TEN 1,000 Hz peak, which could be reflecting olivocochlear activation (Lima da Costa et al., 1997; Guinan, 2006) or middle ear muscle reflex activation (Liberman and Guinan, 1998). However, there is a physiological dilemma with the activation of these feedback circuits, as efferent or middle ear muscle recruitment would produce a decrease in auditory nerve activity, which in turn would decrease efferent and middle ear function. Still, we do not have any better explanation for reductions of the TEN 1,000 Hz peak amplitude during broad-band noise stimulation.

### TEN 1,000 Hz peak and vestibular stimuli

We found an amplitude increase of the TEN 1,000 Hz peak during both caloric stimulation periods (Figure 6). Importantly, we showed that ocular movements alone did not increase the power of the spectral peak at 1,000 Hz, indicating that the increase during caloric tests was not due to nystagmus. Unexpectedly, either warm or cold stimuli caused an increase in the TEN 1,000 Hz peak amplitude, despite the evidence that warm temperatures increase firing rate over the spontaneous level while cold decrease neural responses (Young and Anderson, 1974). One difference between warm and cold stimulation was that the former produced a sustained increase (at least upon the end of our protocol) that was not observed in the latter. One possibility is that the active warming up to body temperature after cold air stimulation might be faster than the cooling down after warm air stimulation, explaining the difference in recovery between cold and warm stimulation.

In addition to cold and warm vestibular stimulation, we performed temperature controls with airflow at 37°C that also produced a small increase in the TEN 1,000 Hz peak (see Figure 6). There is no single answer to elucidate these findings; consequently, we give speculative hypotheses to explain these results. The first hypothesis is that we may have stimulated vestibular afferents with all caloric stimuli, including warm, cold and 37°C degrees. This idea is supported by the fact that two of the five subjects had evoked nystagmus and vertigo using stimulation at 37°C. In addition, in the three included subjects (at 37°C) nystagmus was evaluated by visual inspection with Frenzel goggles. One possibility is that all our subjects stimulated at 37°C could had nystagmus if they had been evaluated with electronystagmography (ENG) or video-oculography (VOG). The lack of ENG or VOG recordings to evaluate the presence of nystagmus is a limitation of our study, that should be addressed in the future. The second hypothesis is that the airflow of the caloric stimulation (independently of the temperature) produced a low intensity acoustic noise in the low frequency band that could modify the amplitude of the TEN 1,000 Hz peak.

### Neural source of the TEN 1,000 Hz peak

Regarding the possible neural sources of the TEN 1,000 Hz peak, we ruled out possible contributions of facial and trigeminal nerves, and we found clear responses to auditory stimuli. Our results with the vestibular stimulation are not conclusive, and more research with other vestibular stimuli (vibration or rotation) should be performed. We propose a mixed origin in humans, with peripheral and central neural contributions, including the auditory nerve, and central auditory pathways. Another possibility is that peripheral and central vestibular pathways are also contributing to this signal. Similar to the auditory brainstem responses, in which far-field potentials can be recorded from the scalp (Jewett et al., 1970; Jewett and Williston, 1971), the origin of the TEN 1,000 Hz peak could involve asynchronous brainstem sources from the auditory pathways, but even from other brain structures not related auditory inputs.

### Possible clinical use of TEN 1,000 Hz peak

To date, ECochG is one of the few techniques that allows a non-invasive functional evaluation of the auditory nerve, mainly focused on CAP measurements. Results show that RW response magnitudes correlate with speech perception outcomes after cochlear implantation in adult (Fitzpatrick et al., 2014; McClellan et al., 2014) and pediatric population (Formeister et al., 2015). Nevertheless, CAP recordings require a synchronizing stimulus (acoustic or electrical) to evoke neural responses, implying difficulties in the case of profound deaf patients, since adequate auditory synchronization could not be achieved. On the other hand, electrical stimulation on the promontory or through cochlear implants are invasive techniques usually performed during ear surgery, which restrict its predictive usefulness of auditory nerve functionality, whereas the measurement of the TEN 1,000 Hz peak may represent a non-invasive option to explore auditory-nerve activity before surgery.

It has also been suggested that the RWN could be employed in the study of tinnitus (McMahon and Patuzzi, 2002; Sendowski et al., 2006), which is the perception of a sound in the absence of any external stimulation (Cazals et al., 1998). Previous studies of the RWN in animals have shown that after administration of salicylate, a chemical that triggers reversible tinnitus in humans (McCabe and Dey, 1965), the broad peak around 900 Hz decreases while a narrow spectral peak around 200 Hz emerges (Snyder and Schreiner, 1987; Martin et al., 1993; Cazals et al., 1998). Similar to these results, a 200 Hz peak component has also been recorded in humans with tinnitus during surgery (Martin, 1995; Feldmeier and Lenarz, 1996). In the present study, we found that masseter muscle activation produced an amplitude increase of frequencies below 800 Hz (Figure 3), probably reflecting EMG activity. As tinnitus pathophysiology can involve hearing loss and/or head and neck injuries (Langguth et al., 2013), we propose that non-invasive measurements of the TEN spectrum in humans could be useful to evaluate tinnitus patients.

## Conclusions

We found a reliable frequency peak at 1,000 Hz in the TEN of humans. The amplitude of this TEN 1,000 Hz peak was modified by acoustic and caloric vestibular stimulation. We propose the TEN 1,000 Hz peak as a potential clinical non-invasive measure for the functional study of the auditory nerve in humans. Future research in bilateral and unilateral deafness subjects and in patients with vestibular function loss will help to unravel the contributions of the vestibular and auditory nerve to the TEN 1,000 Hz peak.

## Author contributions

JP, CD, MB, and PD designed research, analyzed data, and wrote the manuscript; JP, CD, and MB performed research.

### Conflict of interest statement

The authors declare that the research was conducted in the absence of any commercial or financial relationships that could be construed as a potential conflict of interest.
